# Fungal Pathogens Associated with Crown and Root Rot in Wheat-Growing Areas of Northern Kyrgyzstan

**DOI:** 10.3390/jof9010124

**Published:** 2023-01-16

**Authors:** Göksel Özer, İsmail Erper, Şenol Yıldız, Tuğba Bozoğlu, Sezim Zholdoshbekova, Mehtap Alkan, Fatih Tekin, Tair Esenali Uulu, Mustafa İmren, Abdelfattah A. Dababat, Sibel Derviş

**Affiliations:** 1 Department of Plant Protection, Faculty of Agriculture, Bolu Abant Izzet Baysal University, 14030 Bolu, Türkiye; 2 Department of Plant Protection, Faculty of Agriculture, Kyrgyz Turkish Manas University, Bishkek 720044, Kyrgyzstan; 3 Department of Plant Protection, Faculty of Agriculture, Ondokuz Mayis University, 55139 Samsun, Türkiye; 4 International Maize and Wheat Improvement Centre (CIMMYT), P.O. Box 39, Emek, 06170 Ankara, Türkiye; 5 Department of Plant and Animal Production, Vocational School of Kızıltepe, Mardin Artuklu University, 47000 Mardin, Türkiye

**Keywords:** *Triticum* spp., wheat diseases, crown and root rot, pathogenicity

## Abstract

Fungal species associated with crown and root rot diseases in wheat have been extensively studied in many parts of the world. However, no reports on the relative importance and distribution of pathogens associated with wheat crown and root rot in Kyrgyzstan have been published. Hence, fungal species associated with wheat crown/root rot were surveyed in three main wheat production regions in northern Kyrgyzstan. Fungal species were isolated on 1/5 strength potato-dextrose agar amended with streptomycin (0.1 g/L) and chloramphenicol (0.05 g/L). A total of 598 fungal isolates from symptomatic tissues were identified using morphological features of the cultures and conidia, as well as sequence analysis of the nuclear ribosomal internal transcribed spacer (ITS) region, the translation elongation factor 1α (*TEF1*), and the RNA polymerase II beta subunit (*RPB2*) genes. The percentage of fields from which each fungus was isolated and their relative percentage isolation levels were determined. *Bipolaris sorokiniana*, the causal agent of common root rot, was the most prevalent pathogenic species isolated, being isolated from 86.67% of the fields surveyed at a frequency of isolation of 40.64%. *Fusarium* spp. accounted for 53.01% of all isolates and consisted of 12 different species. The most common *Fusarium* species identified was *Fusarium acuminatum*, which was isolated from 70% of the sites surveyed with an isolation frequency of 21.57%, followed by *Fusarium culmorum*, *Fusarium nygamai*, *Fusarium oxysporum*, and *Fusarium equiseti*, all of which had a field incidence of more than 23%. Inoculation tests with 44 isolates representing 17 species on the susceptible *Triticum aestivum* cv. Seri 82 revealed that *Fusarium pseudograminearum* and *F. culmorum* isolates were equally the most virulent pathogens. The widespread distribution of moderately virulent *B. sorokiniana* appears to be a serious threat to wheat culture, limiting yield and quality. With the exception of *F. culmorum*, the remaining *Fusarium* species did not pose a significant threat to wheat production in the surveyed areas because common species, such as *F. acuminatum*, *F. nygamai*, *F. oxysporum*, and *F. equiseti*, were non-pathogenic but infrequent species, such as *Fusarium redolens*, *Fusarium algeriense*, and *F. pseudograminearum*, were highly or moderately virulent. *Curvularia inaequalis*, which was found in three different fields, was mildly virulent. The remaining *Fusarium* species, *Fusarium solani*, *Fusarium proliferatum*, *Fusarium burgessii*, and *Fusarium tricinctum*, as well as *Microdochium bolleyi*, *Microdochium nivale*, and *Macrophomina phaseolina*, were non-pathogenic and considered to be secondary colonizers. The implications of these findings are discussed.

## 1. Introduction

Wheat (*Triticum aestivum* L. and *Triticum durum* Desf.) is the second most important and widely produced staple crop in the world, farmed on 219 million ha of land and producing an annual average of 760.9 million tons in 2020, only trailing behind maize in terms of grain production [1]. Human diets and animal feeds rely heavily on wheat as the primary source of calories (19%) and proteins (20%) [2]. In Central Asia—encompassing Kazakhstan, Uzbekistan, Kyrgyzstan, Tajikistan, and Turkmenistan—wheat is also by far the most significant staple crop [3]. With 629,052 tons of wheat produced on 247,028 ha, Kyrgyzstan is currently the 59th largest producer of wheat in the world [1]. Therefore, the productivity and grain quality of wheat, the crop accounting for 30% of Kyrgyzstan crop sown area [3], are essential to the country’s economic development strategies. Nevertheless, wheat yield in Kyrgyzstan (2.55 tons/ha) is considerably lower than the world average (3.47 tons/ha) [1] due to major biotic and abiotic stress factors. Since the country’s rainfed areas are only found in its mountainous terrain, the majority of Kyrgyzstan’s irrigated lands are situated in its low-lying regions. Water from the Syr Darya and Amu Darya rivers is diverted and used to cultivate lowland agricultural areas where wheat is planted under semi-supplementary irrigation conditions. As a result, the country’s main abiotic stressors are drought and heat stress caused by water scarcity, which is a global issue [3,4]. Stripe or yellow rust produced by *Puccinia striiformis* f. sp. *tritici* is regarded as the most significant foliar disease affecting wheat in Kyrgyzstan [5], despite a general lack of documentation of biotic stress factors. For instance, Dzhunusova et al. (2009) [6] reported that 10–30% of wheat production losses are caused by stripe rust in Kyrgyzstan. There have also been several reports on the genetic differentiation of leaf rust (brown rust) populations [7], as well as varietal screening and resistance to stripe rust, in Kyrgyzstan [5,8]. Fungal populations associated with diseases in wheat underground and crown tissues, on the other hand, have never been studied in comparison to foliar diseases.

Root and stem base rot diseases represent one of the major limiting factors for wheat production, especially under high pathogen pressure [9]. Wheat yields, stands, and grain quality are often decreased by these diseases caused by a range of fungal pathogens [10]. Infection of the root and crown causes constriction of the vascular system, which restricts the absorption and transfer of water and generates whiteheads during the filling phase. Multiple fungal species typically coexist and may cooperate in either a synergistic or competitive manner, influencing their progression and disease-causing abilities. In the largest wheat-producing countries of global significance, field assessments of the crown and root rot pathogens on wheat are regularly reported [11,12,13,14,15,16,17,18,19,20,21]. Various fungal species, including *Bipolaris sorokiniana*, many *Fusarium* species, *Microdochium* spp., *Rhizoctonia* spp., and *Curvularia* spp., exist in mixed populations in wheat according to these studies, but the prevalence and virulence of each species vary across various countries and geographical regions. Common root rot—caused by *B. sorokiniana*—and Fusarium crown rot—caused by numerous *Fusarium* species, primarily *Fusarium pseudograminearum*, *Fusarium culmorum*, and *Fusarium graminearum*—are the most prevalent and significant diseases worldwide [11,20,21,22,23,24]. In China, the world’s largest producer of wheat, *B. sorokiniana* is listed as the major cause of root and crown rot in wheat [11], and it is also acknowledged as one of the most destructive infections of wheat globally [25,26,27]. Similarly, the most prevalent species associated with this disease in Australia [28], North America [12,22], Turkey [19], Azerbaijan [13], and Kazakhstan [21] appear to be *F. pseudograminearum* and *F. culmorum,* which cause the greatest disease severity and reduce grain yield. Discrepancies among the geographical populations of these fungi could be attributed to differences in agroecological zones, farming and management practices, and seasonal environmental conditions in the sampling locations [10,29]. Furthermore, with their complex population dynamics, some species, such as *B. sorokiniana*, *Fusarium avenaceum*, *Fusarium poae*, *F. graminearum*, *F. culmorum*, *Microdochium nivale*, and *Microdochium majus*, which can be seed-borne, soilborne, or residue-borne, cause a variety of diseases in wheat during its development, such as spot blotch, black point, seedling blight, root rot, foot/crown rot, and head blight [26,30,31]. Due to the intricacy of crown and root rot diseases, scientists and cereal producers have failed to control it for decades [32]. In-depth knowledge of pathogen biology and populations, as well as the epidemiological aspects that lead to disease exacerbation, is necessary to combat the disease.

Nothing is known about the prevalence and distribution of diseases in the underground and crown tissues of wheat in Kyrgyzstan. Thus, the current study aimed to (i) identify the fungal species causing root and crown rot in wheat in the main wheat-growing regions of Kyrgyzstan using morphological and molecular methods, including sequencing of the ITS, *TEF1*, and *RPB2* loci; (ii) determine the distribution and frequency of each species; and (iii) evaluate the virulence of each fungal species obtained from wheat roots and crowns. This is the first detailed survey study in Kyrgyzstan that shows pathogen population distribution data from various fields across a wide geographical range.

## 2. Materials and Methods

### 2.1. Protocol for Surveys

**Figure 1:**
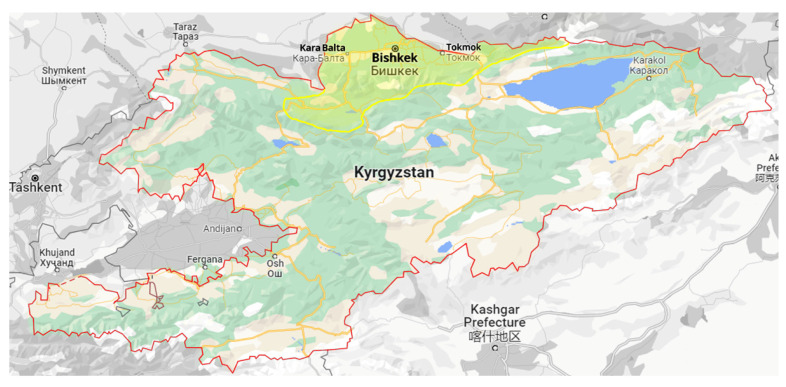
Area of northern Kyrgyzstan surveyed in 2020, marked with yellow coloring.

The findings of this study were derived from a survey of wheat-growing areas in three regions of northern Kyrgyzstan in 2020 (Figure 1). At the crop’s maturity stage and harvesting time, plant samples with characteristic crown and root rot symptoms were collected from 30 wheat fields (10 in the Bishkek, 10 in the Sokuluk, and 10 in the Kara-Balta regions) chosen at random without any prior knowledge of current disease incidence or severity or wheat cultivars. Within the targeted regions, the minimum distance between sampling fields was 5 km. Plants in each field were sampled in 20–30 replicates in a zig-zag pattern at different field sites of 1–2 m^2^ with a minimum distance of 30 m between them, with each sampling site being at least 15 m from the field’s margin. Samples were transported to the lab in paper bags and kept there at room temperature.

### 2.2. Culture Isolation, Preservation, and Morphological Identification

The crown, root, and stem base of the sampled tissues were thoroughly washed under tap water for 15 min before being examined for lesions. Sections of the healthy and symptomatic crown, subcrown, and root tissues of sampled plants were cut into 1 cm lengths. These sections were then surface sterilized with a solution of 75% ethanol for 10 s and 1% sodium hypochlorite for 1–2 min. The sections were then rinsed with sterile water, blotted dry, and placed on 1/5 strength potato-dextrose agar (PDA) (40 g of finely diced potato tubers were boiled in 500 mL distilled water for 30 min, distilled water was added to the filtrated broth to make 1 L, 4 g of dextrose and 15 g of agar were added, and the broth was autoclaved). Streptomycin (0.1 g/L) and chloramphenicol (0.05 g/L), sterilized by filtering through a 0.2 µm Millipore filter (Millipore Corp., Bedford, MA, USA), were added to the PDA after autoclaving to inhibit general bacteria. After 3–7 days of incubation in the dark, filamentous fungal colonies resulting from the sections were sub-cultured on full-strength PDA (200 g diced potato, 20 g dextrose, and 15 g agar) plates and purified using the hyphal tip or the single-spore isolation method under a photoperiod of 12 h of darkness and 12 h of light with a light intensity of 5000 lux at 25 °C. Fusarium-like colonies were also transferred to Spezieller Nährstoffarmer Agar (SNA) (1 g KH_2_PO_4_, 1 g KNO_3_, 0.5 g MgSO_4_·7H_2_O, 0.5 g KCl, 0.2 dextrose, 0.2 sucrose, and 20 g agar in 1 L distilled water) and incubated for 10 days for conidia and chlamydospores production under the same conditions [33]. Fungal isolates were initially identified using morphological and cultural characteristics (microscopic features, colony appearance, and pigmentation) and traditional species identification keys, including description keys for *Fusarium* spp. [33,34,35], *Bipolaris* sp., *Curvularia* sp. [36], *Macrophomina* sp. [37], and *Microdochium* spp. [38]. Some *Fusarium* isolates were challenging to identify at the species level based on morphology alone; hence, all species-identified isolates were subjected to molecular characterization alongside unknown species. All isolates were kept at 4 °C on PDA slants during the studies and stored at −80 °C in vials containing a 15:85 (*v*/*v*) glycerol:water solution for long-term storage.

### 2.3. Molecular Identification

To further confirm the morpho-cultural identification of the fungi, genomic DNA was extracted from all isolates using a DNeasy Blood and Tissue Kit (Qiagen, Hilden, Germany) according to the instructions of the manufacturer. By gently scraping the surfaces of 7 day old PDA cultures incubated at 23 °C, 50–100 mg of fungal mycelia with spores was collected and ground to a powder in liquid nitrogen using a mortar and pestle. The A260/A280 ratio was used to calculate DNA concentration with a DS-11 FX+ nano spectrophotometer (Denovix Inc., Wilmington, DE, USA). The DNA extract was diluted with Tris-EDTA buffer (TE; 10 mM Tris-HCl, 1 mM EDTA, pH 8.0) to 10 ng/L and stored at 20 °C before further analysis.

The nuclear ribosomal internal transcribed spacer (ITS) region for non-*Fusarium* species and the translation elongation factor 1α (*TEF1*) and the RNA polymerase II second largest subunit (*RPB2*) genes for *Fusarium* species were sequenced using primers ITS1 and ITS4 [39], EF1 and EF2 [40], and 5f2 [41] and 7cr [42], respectively. The PCR mixture contained 1× PCR reaction buffer, 1.5-unit Ampliqon TEMPase Hot Start DNA polymerase (Berntsen, Rdovre, Denmark), 0.4 µM of each primer, 0.2 mM of each dNTP, 10 ng template DNA, and sterile milli-Q water up to 50 μL. In a T100 thermal cycler (Bio-Rad Laboratories, Hercules, CA, USA), the PCR amplification for all three loci was carried out with a 15 min initial denaturation at 95 °C, followed by 45 s at 95 °C, 45 s annealing at 54 °C, 90 s extension at 72 °C for 35 cycles, and a 10 min final extension at 72 °C. Sequencing of the PCR products bidirectionally was carried out by Macrogen Inc. (Seoul, Korea) using the same primers.

A Molecular Evolutionary Genetics Analysis (MEGA X) workflow was used across computing platforms to edit DNA sequences, and consensus sequences were calculated manually [43]. Using the BLASTn algorithm (https://blast.ncbi.nlm.nih.gov/Blast.cgi, accessed on 21 September 2022), all sequences were compared to previously published sequences as a template for a homology search to locate each region in GenBank, National Center for Biotechnological Information. The sequences of representative isolates for determining species obtained in this study were deposited in GenBank under accession numbers OP709678-OP709692 for ITS, OP688131-OP688159 for *TEF1*, and OP688160-OP688188 for *RPB2*.

### 2.4. Phylogenetic Analysis

The *Fusarium* isolates from this study, along with additional reference sequences and two outgroups from the GenBank database, were aligned using the MAFFT v.7 online interface (http://mafft.cbrc.jp/alignment/server/, accessed on 21 September 2022) [44] and manually edited with MEGA X. Maximum likelihood (ML) gene trees were inferred separately for the *TEF1* and *RPB2* datasets for *Fusarium* species using the command-line version of IQ-TREE 1.6.7 [45] with an ultrafast bootstrap approximation approach (UFBoot2) implemented with 1000 replicates [46]. The CIPRES Science Gateway V 3.3 was used for the analyses (https://www.phylo.org/, accessed on 21 September 2022).

### 2.5. Fungal Species Frequency in Isolation and Incidence in the Fields

Following species identification of each isolate, the isolation frequency and field incidence of fungal species were estimated. Isolation frequency was calculated by dividing the total number of isolates obtained by the number of fungal isolates obtained per species and expressing the result as a percentage. Individual species incidence in the fields was calculated by dividing the number of locations from which fungal species were recovered by the total number of fields surveyed and expressing the result as a percentage.

### 2.6. Pathogenicity Tests

To evaluate the pathogenicity of the isolates on wheat seedlings under growth room conditions with a 12/12 h light/dark regime at 23 °C, 70 isolates representing 17 species (1 to 5 isolates of each species) were chosen from diverse locations to ensure spatial coverage. For this purpose, bread wheat (*Triticum aestivum* L.) cultivar Seri 82 seeds were treated with 0.5% NaClO for 5 min. The seeds were then placed in plates with a piece of sterile filter paper moistened with water for 3 days to promote germination. A potting mixture of peat (KTS 1, Klasmann-Deilmann, Geeste, Germany), sterile vermiculite, and sterile soil (1:1:1, *v*/*v*/*v*) was placed in plastic pots 17 cm high with a 15 cm diameter. Five identical seedlings were placed on the mixture substrate surface of each pot, and three replicated pots were used for each isolate. *Fusarium* spp. isolates were inoculated by removing ten mycelial plugs from the margin of each isolate’s actively growing PDA plate with a 10 mm diameter sterile cork borer, placing them around the wheat seedlings (two plugs around each seedling), and covering them with the mixture substrate [47]. Control treatments included the same number of sterile agar plugs. To assess the pathogenicity of two dematiaceous fungal species (*B. sorokiniana* and *C. inaequalis*) and *Microdochium* spp., a conidial suspension of each isolate was injected at a density of 250 conidia per gram into the potting mixture used to cover the seedlings [48]. The same volume of sterile distilled water was used to inoculate control seedlings. The pathogenicity of *M. phaseolina* isolates was determined using the colonized wheat kernels method [49], which involved placing ten colonized wheat kernels in contact with wheat seedlings before covering them with the potting mixture. The control group received the same number of autoclaved wheat kernels. Six weeks after incubation, plants were uprooted, cleaned, and examined for lesions or discolorations on the tissues of the root, sub-crown internode, and crown. An index system with a 1–5 scale [50] was used to assess disease symptoms based on the percentage of typical browning/rot at the crown and base of the stem. The mean disease ratings for each isolate were determined with 15 replicated seedlings (3 pots, 5 seedlings per pot). The experiment was carried out once more, and mean scores of 1–2 were considered non-pathogenic (NP) or mildly virulent (MiV) if there were significant differences between treatment and control. Scores of 2–3 were regarded as moderately virulent (MV), while scores greater than 3 were regarded as highly virulent (HV) [51].

Disease severity scores in the pathogenicity tests were analyzed for significance using analysis of variance followed by Tukey’s honestly significant difference (HSD) test at *p* = 0.05 using Statistical Analysis System (SAS Version 9.0; SAS Institute Inc.; Cary, NC, USA).

## 3. Results

In 2020, 598 fungal isolates were isolated from symptomatic wheat samples collected from 30 fields in the wheat-growing regions of northern Kyrgyzstan. Using morphological and molecular techniques, 243 isolates of *B. sorokiniana*, 317 of *Fusarium* spp., 19 of *Curvularia inaequalis*, 11 of *M. phaseolina*, and 8 of *Microdochium* spp. were identified (Table 1). All identified cultures had the general morphological and cultural features (color, size, presence, and shape of the cultures, conidia, conidiophores, septa, and sclerotia) of relevant fungal genera and species based on description keys for *Fusarium* spp. [33,34,35], *Bipolaris* sp., *Curvularia* sp. [36], *Macrophomina* sp. [37], and *Microdochium* spp. [38].

*Bipolaris sorokiniana*, the anamorph of *Cochliobolus sativus*, was the most frequently recovered species (37.75% to 44.39%), accounting for 40.64% of the isolates found in 86.67% of the surveyed fields (Table 1). Another anamorph of *Cochliobolus*, *C. inaequalis*, was represented by 19 isolates from three fields in the Sokuluk and Kara-Balta regions. *M. phaseolina*, *M. bolleyi*, and *M. nivale* were isolated from 23.33%, 6.67%, and 6.67% of the examined fields with isolation frequencies of 1.84%, 0.84%, and 0.50%, respectively. The accession numbers for the representative ITS sequences of the species determined in this study are listed in Table S1.

**Figure 2:**
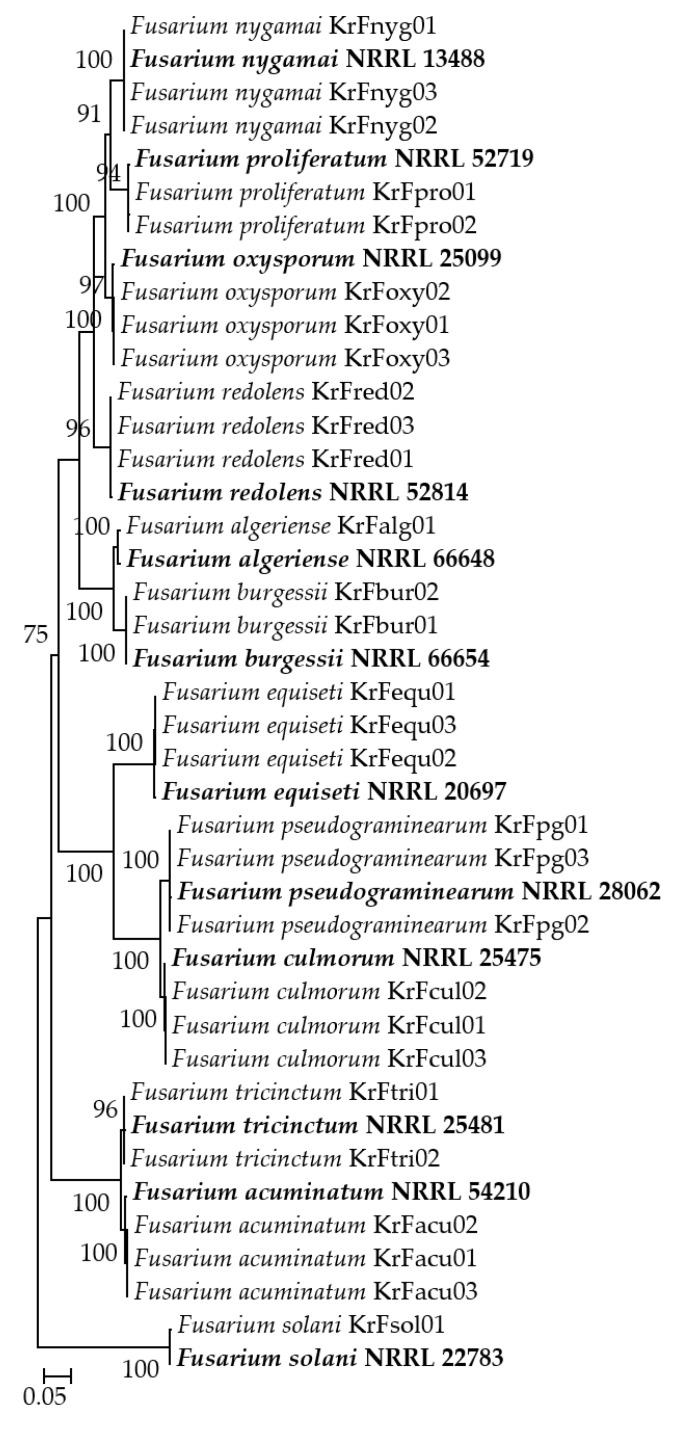
Phylogenetic tree based on maximum likelihood using IQ-TREE with *TEF1* and *RPB2* sequences of *Fusarium* isolates. At each node are the bootstrap values (**left**) and posterior probabilities (**right**). Sequences used as references in the present study are presented in bold.

By comparing them to the published descriptions and DNA sequencing, 317 isolates of *Fusarium* spp., which made up 53.01% of the total number of isolates, were categorized into 12 *Fusarium* species. The *TEF1* and *RPB2* sequences of *Fusarium* spp. isolates had lengths ranging from 636 to 681 bp and 863 to 902 bp, respectively, and were 99–100% identical to the relevant *Fusarium* species in the GenBank database. The accession numbers for the representative *Fusarium* sequences are given in Table S1. Phylogenetic analyses based on the *TEF1* and *RPB2* sequences of *Fusarium* isolates in this study and reference sequences derived from GenBank showed that the isolates belonging to the same species were clearly separated in the dendrogram (Figure 2).

The most frequent *Fusarium* species were *F. acuminatum* and *F. culmorum*, which accounted for 40.69% and 20.82%, respectively, of *Fusarium* spp. isolates. These two species were followed by *F. nygamai*, *F. equiseti*, and *F. oxysporum*, which accounted for 13.88%, 8.20%, and 5.99%, respectively. *Fusarium acuminatum* was found in 70.00% of the surveyed fields, *F. culmorum* in 46.67%, *F. nygamai* and *F. oxysporum* in 30.00%, and *F. equisetum* in 23.33%. With the exception of *F. nygamai*, all of the aforementioned *Fusarium* species were present in all three of the surveyed regions; however, the occurrence of each species varied by region. Less frequently occurring *Fusarium* species included *F. pseudograminearum* and *F. redolens*, with frequencies of 5.36% and 2.52%, respectively. Two *F. proliferatum* isolates were only found in one field in Bishkek, and two isolates of *F. tricinctum* and *F. burgessii* were in one and two fields in Sokuluk, respectively. *Fusarium solani* and *F. algeriense* were represented by one isolate from Kara-Balta and Sokuluk. *Bipolaris sorokiniana, F. acuminatum,* and *F. culmorum* were commonly found coexisting in one field, as their 243, 129, and 66 isolates were identified in 86.67%, 70.00%, and 46.67% of the fields surveyed, respectively.

The results of the pathogenicity tests showed that the isolates of *F. pseudograminearum* and *F. culmorum* were equally the most virulent pathogens (*p* < 0.05), with mean crown rot severity scores of 3.67 and 3.62, respectively (Table 2). *Bipolaris sorokiniana*, with a mean severity of 2.78, was moderately virulent on the crowns and roots, causing necrosis or rot at levels comparable to some *F. algeriense*-inoculated plants. Similarly, *F. redolens* isolates had a mean crown rot severity of 2.23 and were moderately virulent, though not as severe as the aforementioned species, and they were similar to some *F. algeriense*-inoculated plants. Accordingly, *F. algeriense* was as virulent as *B. sorokiniana* and *F. redolens*, with a mean crown rot severity of 2.22. The isolates of *C. inaequalis* had a mean severity score of 1.67 and were mildly virulent but had some similarities to some *F. algeriense*-inoculated plants. Despite having a disease severity score of less than 2, they showed significant differences from the control and other non-pathogens at *p* = 0.05. The remaining *Fusarium* isolates—*F. nygamai*, *F. solani*, *F. oxysporum*, *F. proliferatum*, *F. burgessii*, *F. tricinctum*, *F. acuminatum*, and *F. equiseti*—as well as both *Microdochium* species—*M. bolleyi* and *M. nivale*—and *M. phaseolina* were non-pathogenic on wheat seedlings, with no statistically significant differences from control plants.

## 4. Discussion

Wheat is grown in a variety of geographical regions, environments, and production systems around the world. In Kyrgyzstan, the 59th largest producer of wheat, no research has ever been undertaken on the presence and distribution of fungal infections in wheat subterranean and crown tissues. The findings of a systematic survey of pathogens associated with wheat crown rot in northern Kyrgyzstan are presented in this article. *Bipolaris sorokiniana* was identified as the primary pathogen of wheat in this part of the country, with a high field incidence (86.67%) and isolation frequency (40.64%) from roots and crowns. The results also demonstrated the widespread distribution of Fusarium crown rot in this area, as well as the presence of 12 *Fusarium* species on the bases of wheat stems. The two species that were most prevalent in the majority of the sampling regions were *F. acuminatum* and *F. culmorum*. Although the frequency of *F. acuminatum* isolation was only 21.57%, it was found in 70.00% of the surveyed fields. These high *B. sorokiniana* and *F. acuminatum* ratios are comparable to those found in Colorado and Wyoming [52], Mississippi [53], Montana [14], western Canada [54], northern China [10], North Dakota [55], and Kazakhstan [21]. Similarly, *F. culmorum* was identified in 46.67% of the investigated fields, with an 11.04% isolation frequency. *Fusarium culmorum*, ranking third in field incidence and isolation frequency in this study, has been identified as a major component of the most common and destructive *Fusarium* species in numerous surveys conducted in various wheat-growing regions [14,18,19,55,56,57,58,59,60]. *Fusarium nygamai* was another common *Fusarium* species, with 44 isolates collected from eight fields in Bishkek and one in Sokuluk—representing 30.00% of the sites surveyed—and an isolation frequency of 7.36%. *Fusarium nygamai* was previously isolated from stored wheat samples from moderate to warm climates [61] and was found to be one of the most common *Fusarium* species in wheat root and stem tissues in Iran [62]. It was also isolated from wheat roots in the Iraqi province of Basra [63]. *Fusarium equiseti*, which ranked fifth in terms of field incidence and isolation frequency, has been reported as a dominant fungus associated with crown and root rot in wheat in Azerbaijan [13], North Dakota [55], Saskatchewan [64], Mississippi [53], Italy [65], Canada [54], and Turkey [19,66], which confirms our findings. *Fusarium oxysporum* and *M. phaseolina* were also found in 30.00% and 23.33% of the sampling fields, respectively, with isolation rates of 3.18% and 1.84%. A high frequency of *F. oxysporum* isolation has also been reported from Western Canada [54], Serbia [67], and Kazakhstan [21], which is consistent with our observations. The only report of *M. phaseolina* on symptomatic wheat roots came from Kazakhstan, where 3.08% field incidence was reported [21]. The relatively low field incidence (16.67%) and isolation frequency (2.84%) of *F. pseudograminearum* differed from those previously reported for crown and root rot fungi in Australia [28], North America [12], and Turkey [19]. This could be related to the cold temperatures in the surveyed regions. *Fusarium culmorum* is thought to prefer cool and semiarid climates, while *F. pseudograminearum* prefers slightly warmer climates [56]. In North Dakota, for example, cooler years produced higher *F. culmorum* isolation ratios than warmer years, while warmer years produced higher *F. pseudograminearum* isolation ratios [55]. Isolates of *F. redolens*, previously identified as a wheat pathogen in Canada [68], Turkey [69], and Kazakhstan [21], were found in 13.33% of the fields sampled (only in Bishkek). *Curvularia inaequalis* isolates, an anamorph of *Cochliobolus*, were discovered in two fields in the Sokuluk region and one field in Kara-Balta. This species has also been found sporadically in Azerbaijan [13,51] and Kazakhstan [21], which is consistent with the current results. These results suggest that the distribution of *F. nygamai*, *F. redolens*, and *C. inaequalis* may be attributable to their environmental adaption. Less frequently occurring fungal species included *M. bolleyi*, *M. nivale*, *F. proliferatum*, *F. burgessii*, *F. tricinctum*, *F. solani*, and *F. algeriense*. With the exception of *F. burgessii*, these species are frequently isolated from wheat crown and stem tissues all over the world. For instance, *M. bolleyi*, along with *F. graminearum*, *F. avenaceum*, and *F. tricinctum*, was the most frequently isolated species from winter wheat affected by foot and crown rot in New York [70]. In Saskatchewan, Canada, *M. bolleyi* was isolated from discolored crown tissue of common and durum wheat with the second highest percentage of isolation and found in the second highest number of fields after *B. sorokiniana* [64]. The same species was also isolated from the root and crown of soft red winter wheat in Mississippi [53]. *Microdochium nivale,* formerly known as *F. nivale*, was isolated from discolored crown tissues, leaf sheaths, stem bases, and roots of bread and/or durum wheat in the United Kingdom [58], Turkey [18], Lithuania [71], Algeria [60,72], and China [73]. The previous stem base disease surveys found low frequencies of *F. proliferatum* isolation in Mexico [74], Turkey [18,19], China [10], Azerbaijan [13], and Iran [75], similarly to our research. *Fusarium tricinctum* was isolated from wheat roots and crowns in New York [70] and Turkey [18,19], and it was the dominant species detected on stem bases in Germany [76]. This is the first time *F. burgessii* has been found on wheat roots or crowns.

In general, the pathogenicity results presented here are consistent with those presented in other studies conducted in wheat-growing regions around the world. Among all the species, *F. pseudograminearum* and *F. culmorum* were equally the most virulent pathogens that killed seedlings, which is consistent with previous findings [12,13,19,20,21,22,28,77,78]. Following them, *B. sorokiniana*, *F. redolens,* and *F. algeriense* were found to be moderately virulent on the crowns and roots of the bread wheat cultivar Seri 82. *Bipolaris sorokiniana*, on the other hand, was more virulent than the others. *Bipolaris sorokiniana* is widely regarded as one of the most damaging wheat pathogens in the world [10,21,25,26,27,51]. *Fusarium redolens* was equally as virulent as *F. algeriense* but more virulent than *C. inaequalis. Fusarium redolens* has only been identified as a wheat pathogen in Canada [68], Turkey [19], and Kazakhstan [21]. Previously, *F. algeriense* was found as pathogenic on durum wheat in Algeria [35], as well as bread wheat in Azerbaijan [13] and Kyrgyzstan [79]. *Curvularia inaequalis* was also associated with crown and root rot, albeit to a lesser extent and as a mildly virulent pathogen, which is consistent with previous research [13,21,51].

The remaining fungal species within the research—*F. nygamai*, *F. solani*, *F. oxysporum*, *F. proliferatum*, *F. burgessii*, *F. tricinctum*, *F. acuminatum*, *F. equiseti*, *M. bolleyi*, *M. nivale*, and *M. phaseolina*—were non-pathogenic on wheat seedlings. The findings for non-pathogenic species presented here are somewhat consistent with those from other studies conducted in wheat-producing countries. *Fusarium nygamai*, for instance, caused Fusarium root rot with the lowest disease severity among the *Fusarium* species obtained [63]. In Kazakhstan, *M. phaseolina* was also identified as a non-pathogenic species [21], which is similar to our findings. In another study, *F. oxysporum* and *F. solani* isolates were non-pathogenic on Alsen and ND652 wheat genotypes, and both species were thought to be secondary invaders isolated from dead tissues of infected root samples [55]. When the outer layer of root rot samples was removed for fungal isolation, the frequency of *F. oxysporum* isolation was cut in half. As a result, the authors proposed removing the outer layer of infected tissue during pathogen isolation in order to isolate the fungal species that are truly associated with root rot disease and reduce the possibility of secondary invaders. On the other hand, in the same study, *F. acuminatum*, *F. equiseti*, and *F. redolens* were pathogenic, causing infections in seedlings of the two wheat genotypes (ND652 and Alsen) [55]. Similar to our research, *F. oxysporum*, *F. equiseti*, *F. solani*, *F. tricinctum*, *F. acuminatum*, and *F. proliferatum* were non-pathogenic in durum wheat [19]. In contrast to these reports, the roots of the most widely planted spring wheat cultivar in Egypt, Sakha 69, were rotted by *M. phaseolina*, and some isolates of *F. oxysporum* were capable of killing plants [80]. In Iran, *F. pseudograminearum*, *F. culmorum*, and *F. solani* had the highest disease index, while *F. equiseti* had the lowest crown and root rot severity [75]. In test tube cultures, *F. acuminatum*, *F. solani*, *F. equiseti*, and *M. bolleyi* caused slight to moderate orange to light-brown discoloration of the crown and seminal roots [53]. In the greenhouse, *F. acuminatum* reduced seedling height, emergence, and root and shoot dry weights. *M. bolleyi* reduced root fresh and dry weight, plant emergence, and shoot dry weight [53]. The effects of *F. tricinctum* and *M. bolleyi* inoculation were less severe in greenhouse experiments compared to the other species obtained, though reductions in growth and yield were observed at high *F. tricinctum* inoculum levels [70]. In field studies, *M. bolleyi* and *F. tricinctum* had no effects on wheat growth or yield [70]. *Fusarium acuminatum*, *F. tricinctum*, *F. proliferatum*, and *F. pseudograminearum* were found to be capable of producing crown rot under laboratory conditions in Australia [28]. *Microdochium bolleyi* has also been proposed as a biocontrol agent for cereal stem base pathogens [81] and as an endophytic fungus [82]. As a result, it is likely that this non-pathogenic *Fusarium* species colonizes wheat stem bases latently but cannot cause disease. Pathogenicity tests on wheat crown, leaves, and heads revealed that *M. nivale* isolates recovered from both durum and bread wheat crowns were capable of causing infection in durum wheat crowns, leaves, and heads, indicating the nonhost specialization of this species [72]. Demirci and Dane [83] investigated the cause of crown and root rots in the winter wheat Kirik and found that *M. nivale* was the most virulent pathogen under greenhouse conditions, while *F. acuminatum*, *F. equiseti*, *F. oxysporum*, and *F. solani* were only slightly virulent. Furthermore, *M. nivale* isolates were found to be pathogenic in wheat, causing root rot/crown rots/seedling blight in Turkey [18], Lithuania [71], Algeria [72], and China [73]. *Fusarium proliferatum* was one of the two main species causing root rot in wheat in Bajio, Mexico [74]. Similar to our findings, the same species was not found as pathogenic in wheat in Turkey [18,19], China [10], and Azerbaijan [13].

The high field incidence and isolation frequency of *Fusarium culmorum* and the presence of *F. pseudograminearum*, *F. tricinctum*, and *M. nivale* in the surveyed fields may be especially important if they result in Fusarium head blight, which begins at anthesis and spreads until grain harvest, contaminating grain with mycotoxins [30,31,60,84,85]. *Bipolaris sorokiniana* is the causative agent of wheat diseases such as common root rot, spot blotch, seedling blight, and black point, attacking all wheat organs, such as roots, crowns, stems, leaves, and kernels [26]. In warmer growing areas, the fungus is one of the most serious foliar disease constraints, causing significant yield losses [25]. As each of these diseases is managed differently, the presence of these species must be considered when developing a control strategy.

A correct species diagnosis of fungi requires accurate multiphase identification approaches based on morphological and multilocus analysis, as well as phylogenetic analyses. The identification links it to information regarding its host range, geographic dispersion, and ability to produce toxins [86]. This is especially important when it comes to publishing peer-reviewed disease reports, where imprecise and/or incorrect identifications erode public knowledge. The translation elongation factor-1α (*TEF1)* [40] and *RPB2* genes [87] used in this study are the most commonly used genes in the phylogenetic analyses of *Fusarium* species, with *TEF1* serving as a de facto barcode for the genus, though numerous other genes have also been used [86,88,89,90,91]. Using sequencing analysis of each locus and phylogenetic analysis of two genomic loci, all *Fusarium* isolates in the present investigation were correctly identified. This provided methods for rapidly differentiating various fungal species found on wheat roots and crowns, with each species clustering with its associated isolates, as supported by a bootstrap value ranging from 98 to 100%. The two phylogenetically informative genes *TEF1* and *RPB2* have been reported to resolve at or near the species level in all *Fusarium* species [86]. There are also some contradictory reports. For instance, ambiguity in the *TEF1* sequences have been discovered, which prevents differences in this region from unequivocally distinguishing *Fusarium* species from one another [92]. Furthermore, these two genes have some specific applications. For example, in addition to a previously discovered SNP polymorphism in *TEF1* [93], phylogenetic data revealed an SNP polymorphism between fumonisin-producing and nonproducing strains in *RPB2* [94].

## 5. Conclusions

This is the first comprehensive study of the fungi responsible for crown and root rot of wheat conducted in the main wheat-growing areas of northern Kyrgyzstan, and it used morphological and molecular methods to identify the isolates of 17 fungal species, including *TEF1* and *RPB2* loci sequencing. The most commonly isolated species, *B. sorokiniana,* was moderately virulent. The second most frequently isolated fungus was *F. acuminatum*, which was followed by *F. culmorum*, *F. nygamai*, *F. oxysporum*, and *F. equiseti*, all of which had a field incidence of more than 23%. With the exception of *F. culmorum*, the remaining species were non-pathogenic and did not constitute a significant threat to wheat production in the examined locations. However, *F. culmorum*, along with *F. pseudograminearum*, was highly virulent, but *F. pseudograminearum* was relatively infrequent. Despite their infrequency, the species *F. redolens* and *F. algeriense* were moderately virulent, and *C. inaequalis* was a mildly virulent pathogen. Other fungal species included *F. oxysporum*, *M. phaseolina, M. bolleyi*, *M. nivale*, *F. proliferatum*, *F. burgessii*, *F. tricinctum*, and *F. solani*, but they were non-pathogenic in the bread wheat cultivar Seri 82 seedlings. Previous reports on the pathogenicity of some of these fungi in wheat are inconsistent, highlighting the need for additional pathogenicity tests on different cultivars and combined inoculation with the other pathogens.

With the exception of *F. algeriense*, this is the first report of all the fungal species on wheat, some of which are pathogenic. The results of this study illustrate that crown and root rot pathogens are prevalent in wheat-growing fields in northern Kyrgyzstan. This means that similar studies are needed to account for changing pathogen compositions associated with crown and root diseases in other regions of the country that grow wheat. This research will contribute to the formulation of guidelines for the management of root and crown rot fungi in wheat across diverse agronomic zones in Kyrgyzstan in order to keep pathogenic species under control.

## Figures and Tables

**Table 1:** The distribution of fungal species associated with wheat crown/root rot across three different wheat-growing regions in northern Kyrgyzstan.

Species *	Bishkek			Sokuluk			Kara-Balta			Total			
	NF	NI	IF	NF	NI	IF	NF	NI	IF	NF	NI	FI	IF
*Bipolaris sorokiniana*	9	87	44.39	8	77	37.75	9	79	39.90	26	243	86.67	40.64
*Fusarium acuminatum*	5	31	15.82	8	51	25.00	8	47	23.74	21	129	70.00	21.57
*Fusarium culmorum*	3	11	5.61	6	25	12.25	5	30	15.15	14	66	46.67	11.04
*Fusarium nygamai*	8	41	20.92	1	3	1.47	0	0	0.00	9	44	30.00	7.36
*Fusarium equiseti*	3	6	3.06	1	5	2.45	3	15	7.58	7	26	23.33	4.35
*Fusarium oxysporum*	2	5	2.55	2	6	2.94	5	8	4.04	9	19	30.00	3.18
*Curvularia inaequalis*	0	0	0.00	2	13	6.37	1	6	3.03	3	19	10.00	3.18
*Fusarium pseudograminearum*	0	0	0.00	3	8	3.92	2	9	4.55	5	17	16.67	2.84
*Macrophomina phaseolina*	1	2	1.02	4	6	2.94	2	3	1.52	7	11	23.33	1.84
*Fusarium redolens*	4	8	4.08	0	0	0.00	0	0	0.00	4	8	13.33	1.34
*Microdochium bolleyi*	1	3	1.53	1	2	0.98	0	0	0.00	2	5	6.67	0.84
*Microdochium nivale*	0	0	0.00	2	3	1.47	0	0	0.00	2	3	6.67	0.50
*Fusarium proliferatum*	1	2	1.02	0	0	0.00	0	0	0.00	1	2	3.33	0.33
*Fusarium burgessii*	0	0	0.00	2	2	0.98	0	0	0.00	2	2	6.67	0.33
*Fusarium tricinctum*	0	0	0.00	1	2	0.98	0	0	0.00	1	2	3.33	0.33
*Fusarium solani*	0	0	0.00	0	0	0.00	1	1	0.51	1	1	3.33	0.17
*Fusarium algeriense*	0	0	0.00	1	1	0.49	0	0	0.00	1	1	3.33	0.17
Total	10	196	100	10	204	100	10	198	100	30	598	100	100

* NF = Number of fields where individual species were identified; NI = number of isolates; IF = isolation frequency (%); FI = field incidence (%). The species row is arranged in descending order based on the total number of isolates obtained.

**Table 2:** The pathogenicity of the fungal species identified in the present study.

Species	Number of Isolates	Average Severity Index *	Average Disease Severity (%) ***	Virulence Category ****
*Fusarium pseudograminearum*	3	3.67 ± 0.49 a **	73.33	HV
*Fusarium culmorum*	3	3.62 ± 0.51 a	72.33	HV
*Bipolaris sorokiniana*	3	2.78 ± 0.39 b	55.50	MV
*Fusarium redolens*	3	2.23 ± 0.28 c	44.67	MV
*Fusarium algeriense*	1	2.22 ± 0.21 bd	44.50	MV
*Curvularia inaequalis*	3	1.67 ± 0.26 de	33.33	MiV
*Fusarium nygamai*	3	1.43 ± 0.23 e	–	NP
*Fusarium solani*	1	1.42 ± 0.27 e	–	NP
*Microdochium bolleyi*	3	1.42 ± 0.37 e	–	NP
*Fusarium oxysporum*	3	1.40 ± 0.15 e	–	NP
*Fusarium proliferatum*	2	1.39 ± 0.21 e	–	NP
*Macrophomina phaseolina*	3	1.37 ± 0.24 e	–	NP
*Microdochium nivale*	3	1.32 ± 0.26 e	–	NP
*Fusarium burgessii*	2	1.29 ± 0.15 e	–	NP
*Fusarium tricinctum*	2	1.28 ± 0.18 e	–	NP
*Fusarium acuminatum*	3	1.26 ± 0.23 e	–	NP
*Fusarium equiseti*	3	1.25 ± 0.16 e	–	NP

The table rows are ordered from the highest to the lowest disease severity scores. * Standard deviation from the mean of the values belonging to all isolates; ** values followed by the same letter are significantly different among isolates based on Tukey’s HSD at *p* = 0.05; *** disease severity was not calculated for the species considered non-pathogenic; **** HV: highly virulent, MV: moderately virulent, MiV: mildly virulent, NP: non-pathogenic. “–” = No statistically significant differences from control plants.

## Data Availability

All relevant data generated or analyzed during this study are included in this manuscript.

## Supplementary Materials

The following supporting information can be downloaded at: https://www.mdpi.com/article/10.3390/jof9010124/s1, Table S1: GenBank Accession numbers of the representative isolates obtained in this study.

## References

[1] FAOSTAT Food and Agriculture Organization Statistical Database. http://www.fao.org/faostat/en/#data/QC.

[2] Shiferaw B., Smale M., Braun H.J., Duveiller E., Reynolds M., Muricho G. (2013). Crops that feed the world 10. Past successes and future challenges to the role played by wheat in global food security. Food Secur..

[3] Li Z., Fang G., Chen Y., Duan W., Mukanov Y. (2020). Agricultural water demands in Central Asia under 1.5 C and 2.0 C global warming. Agric. Water Manag..

[4] Park S., Lim C.H., Kim S.J., Isaev E., Choi S.E., Lee S.D., Lee W.K. (2021). Assessing climate change impact on cropland suitability in Kyrgyzstan: Where are potential high-quality cropland and the way to the future. Agronomy.

[5] Ziyaev Z.M., Sharma R.C., Nazari K., Morgounov A.I., Amanov A.A., Ziyadullaev Z.F., Alikulov S.M. (2011). Improving wheat stripe rust resistance in Central Asia and the Caucasus. Euphytica.

[6] Dzhunusova M., Yahyaoui A., Morgounov A.I., Jyldyz E. (2006). Resistance of international winter wheat germplasm to yellow rust. Proceedings of the Abstracts of the 3rd Regional Yellow Rust Conference for Central and West Asia and North Africa.

[7] Kolmer J.A., Ordoñez M.E. (2007). Genetic differentiation of *Puccinia triticina* populations in Central Asia and the Caucasus. Phytopathology.

[8] Garadagi S.M. (1976). Resistance to yellow rust in some wheat varieties. Azarb Elmi-Tadg. Akincil Inst. Asar. Temat Macmuasi..

[9] Poole G.J., Harries M., Hüberli D., Miyan S., MacLeod W.J., Lawes R., McKay A. (2015). Predicting cereal root disease in Western Australia using soil DNA and environmental parameters. Phytopathology.

[10] Bockus W.W., Bowden R.L., Hunger R.M., Morrill W.L., Murray T.D., Smiley R.W. (2010). Compendium of Wheat Diseases and Pests.

[11] Xu F., Yang G., Wang J., Song Y., Liu L., Zhao K., Li Y., Han Z. (2018). Spatial distribution of root and crown rot fungi associated with winter wheat in the North China Plain and its relationship with climate variables. Front. Microbiol..

[12] Smiley R.W., Gourlie J.A., Easley S.A., Patterson L.M. (2005). Pathogenicity of fungi associated with the wheat crown rot complex in Oregon and Washington. Plant Dis..

[13] Özer G., Paulitz T.C., İmren M., Alkan M., Muminjanov H., Dababat A.A. (2020). Identity and pathogenicity of fungi associated with crown and root rot of dryland winter wheat in Azerbaijan. Plant Dis..

[14] Moya-Elizondo E.A., Rew L.J., Jacobsen B.J., Hogg A.C., Dyer A.T. (2011). Distribution and prevalence of Fusarium crown rot and common root rot pathogens of wheat in Montana. Plant Dis..

[15] Moya-Elizondo E., Arismendi N., Castro M.P., Doussoulin H. (2015). Distribution and prevalence of crown rot pathogens affecting wheat crops in southern Chile. Chil. J. Agric. Res..

[16] Alahmad S., Simpfendorfer S., Bentley A.R. (2018). Crown rot of wheat in Australia: *Fusarium pseudograminearum* taxonomy, population biology and disease management. Australas. Plant Pathol..

[17] Paulitz T.C., Smiley R.W., Cook R.J. (2002). Insights into the prevalence and management of soilborne cereal pathogens under direct seeding in the Pacific Northwest, USA. Can. J. Plant Pathol..

[18] Tunali B., Nicol J.M., Hodson D., Uçkun Z., Büyük O., Erdurmuş D. (2008). Root and crown rot fungi associated with spring, facultative, and winter wheat in Turkey. Plant Dis..

[19] Shikur Gebremariam E., Sharma-Poudyal D., Paulitz T.C. (2018). Identity and pathogenicity of *Fusarium* species associated with crown rot on wheat (*Triticum* spp.) in Turkey. Eur. J. Plant Pathol..

[20] Zhou H., He X., Wang S., Ma Q., Sun B., Ding S., Chen L., Zhang M., Li H. (2019). Diversity of the *Fusarium* pathogens associated with crown rot in the Huanghuai wheat-growing region of China. Environ. Microbiol..

[21] Bozoğlu T., Derviş S., Imren M., Amer M., Özdemir F., Paulitz T.C., Morgounov A., Dababat A.A., Özer G. (2022). Fungal Pathogens Associated with Crown and Root Rot of Wheat in Central, Eastern, and Southeastern Kazakhstan. J. Fungi.

[22] Dyer A.T., Johnston R.H., Hogg A.C., Johnston J.A. (2009). Comparison of pathogenicity of the Fusarium crown rot (FCR) complex (*F. culmorum*, *F. pseudograminearum* and *F. graminearum*) on hard red spring and durum wheat. Eur. J. Plant Pathol..

[23] Cook R.J., Bockus W.W., Bowden R.L., Hunger R.M., Morrill W.L., Murray T.D., Smiley R.W. (2010). Fusarium root, crown, and foot rots and associated seedling diseases. Compendium of Wheat Diseases and Pests.

[24] Fernandez M.R., Conner R.L. (2011). Root and crown rot of wheat. Prairie Soils Crops J..

[25] Kumar J., Schäfer P., Hückelhoven R., Langen G., Baltruschat H., Stein E. (2002). *Bipolaris sorokiniana*, a cereal pathogen of global concern: Cytological and molecular approaches towards better control. Mol. Plant Pathol..

[26] Al-Sadi A.M. (2021). *Bipolaris sorokiniana*-induced black point, common root rot, and spot blotch diseases of wheat: A review. Front. Cell. Infect. Microbiol..

[27] Acharya K., Dutta A.K., Pradhan P. (2011). *Bipolaris sorokiniana* (Sacc.) Shoem.: The most destructive wheat fungal pathogen in the warmer areas. Aust. J. Crop Sci..

[28] Akinsanmi O.A., Mitter V., Simpfendorfer S., Backhouse D., Chakraborty S. (2004). Identity and pathogenicity of *Fusarium* spp. isolated from wheat fields in Queensland and northern New South Wales. Aust. J. Agric. Res..

[29] Anderson W.K., Garlinge J.R. (2000). The Wheat Book: Principles and Practice. Agric. West. Aust. Bull..

[30] Scherm B., Balmas V., Spanu F., Pani G., Delogu G., Pasquali M., Migheli Q. (2013). *Fusarium culmorum*: Causal agent of foot and root rot and head blight on wheat. Mol. Plant Pathol..

[31] Karlsson I., Persson P., Friberg H. (2021). Fusarium head blight from a microbiome Perspective. Front. Microbiol..

[32] Pérez-Méndez N., Miguel-Rojas C., Jimenez-Berni J.A., Gomez-Candon D., Pérez-de-Luque A., Fereres E., Catala-Forner M., Villegas D., Sillero J.C. (2022). Plant breeding and management strategies to minimize the impact of water scarcity and biotic stress in cereal crops under Mediterranean conditions. Agronomy.

[33] Leslie J.F., Summerell B.A. (2006). The Fusarium Laboratory Manual.

[34] Laurence M.H., Summerell B.A., Burgess L.W., Liew E.C.Y. (2011). *Fusarium burgessii* sp. nov. representing a novel lineage in the genus *Fusarium*. Fungal Divers..

[35] Laraba I., Keddad A., Boureghda H., Abdallah N., Vaughan M.M., Proctor R.H., Busman M., O’Donnell K. (2017). *Fusarium algeriense*, sp. nov., a novel toxigenic crown rot pathogen of durum wheat from Algeria is nested in the *Fusarium burgessii* species complex. Mycologia.

[36] Sivanesan A. (1987). Graminicolous species of *Bipolaris*, *Curvularia*, *Drechslera*, *Exserohilum* and their teleomorphs. Mycologia.

[37] Holliday P., Punithalingam E. (1970). *Macrophomina phaseolina*. C.M.I. Descr. Pathog. Fungi Bact..

[38] Glynn N.C., Hare M.C., Parry D.W., Edwards S.G. (2005). Phylogenetic analysis of *EF-1 alpha* gene sequences from isolates of *Microdochium nivale* leads to elevation of varieties *majus* and *nivale* to species status. Mycol. Res..

[39] White T.J., Bruns T., Lee S., Taylor J., Innis M.A., Gelfand D.H., Sninsky J.J., White T.J. (1990). Amplification and direct sequencing of fungal ribosomal RNA genes for phylogenetics. PCR Protocols, Guide to Methods and Applications.

[40] O’Donnell K., Cigelnik E., Nirenberg H.I. (1998). Molecular systematics and phylogeography of the *Gibberella fujikuroi* species complex. Mycologia.

[41] Reeb V., Lutzoni F., Roux C. (2004). Contribution of *RPB*2 to multilocus phylogenetic studies of the euascomycetes (Pezizomycotina, Fungi) with special emphasis on the lichen-forming Acarosporaceae and evolution of polyspory. Mol. Phylogenet. Evol..

[42] Liu Y.J., Whelen S., Hall B.D. (1999). Phylogenetic relationships among ascomycetes: Evidence from an RNA polymerse II subunit. Mol. Biol. Evol..

[43] Kumar S., Stecher G., Li M., Knyaz C., Tamura K. (2018). MEGA X: Molecular Evolutionary Genetics Analysis across computing platforms. Mol. Biol. Evol..

[44] Katoh K., Rozewicki J., Yamada K.D. (2019). MAFFT online service: Multiple sequence alignment, interactive sequence choice and visualization. Brief. Bioinform..

[45] Nguyen L.T., Schmidt H.A., Von Haeseler A., Minh B.Q. (2015). IQTREE: A fast and effective stochastic algorithm for estimating maximum likelihood phylogenies. Mol. Biol. Evol..

[46] Hoang D.T., Chernomor O., von Haeseler A., Minh B.Q., Vinh L.S. (2018). UFBoot2: Improving the ultrafast bootstrap approximation. Mol. Biol. Evol..

[47] Özer G., İmren M., Paulitz T.C., Bayraktar H., Muminjanov H., Dababat A.A. (2020). First report of crown rot caused by *Fusarium algeriense* on wheat in Azerbaijan. Plant Dis..

[48] Duczek L.J., Verma P.R., Spurr D.T. (1985). Effect of inoculum density of *Cochliobolus sativus* on common root rot of wheat and barley. Can. J. Plant Pathol..

[49] Demirci E. (1997). Some hosts of *Macrophomina phaseolina* (Tassi) Goid. in Erzincan province. Atatürk Üniv. Ziraat Fak. Derg..

[50] Wildermuth G.B., McNamara R.B. (1994). Testing wheat seedlings for resistance to crown rot caused by *Fusarium graminearum* Group 1. Plant Dis..

[51] Özer G., Imren M., Alkan M., Paulitz T.C., Bayraktar H., Palacıoğlu G., Mehdiyev I., Muminjanov H., Dababat A.A. (2020). Molecular and pathogenic characterization of *Cochliobolus* anamorphs associated with common root rot of wheat in Azerbaijan. Phytopathol. Mediterr..

[52] Hill J.P., Fernandez J.A., McShane M.S. (1983). Fungi associated with common root rot of winter wheat in Colorado and Wyoming. Plant Dis..

[53] Gonzales M.S., Trevathan L.E. (2000). Identity and pathogenicity of fungi associated with root and crown rot of soft red winter wheat grown on the upper coastal plain land resource area of Mississippi. J. Phytopathol..

[54] Fernandez M.R., Fox S.L., Hucl P., Singh A.K., Stevenson F.C. (2014). Root rot severity and fungal populations in spring common, durum and spelt wheat, and Kamut grown under organic management in western Canada. Can. J. Plant Sci..

[55] Shrestha S., Poudel R.S., Zhong S. (2021). Identification of Fungal Species Associated with Crown and Root Rots of Wheat and Evaluation of Plant Reactions to the Pathogens in North Dakota. Plant Dis..

[56] Cook R.J., Nelson P.E., Tousson T.A., Cook R.J. (1981). Fusarium Diseases of Wheat and Other Small Grains in North America.

[57] Smiley R.W., Patterson L.M. (1996). Pathogenic fungi associated with Fusarium foot rot of winter wheat in the semiarid Pacific Northwest. Plant Dis..

[58] Pettitt T., Xu X., Parry D. (2003). Association of *Fusarium* species in the wheat stem rot complex. Eur. J. Plant Pathol..

[59] Backhouse D., Abubakar A.A., Burgess L.W., Dennisc J.I., Hollaway G.J., Wildermuth G.B., Henry F.J. (2004). Survey of *Fusarium* species associated with crown rot of wheat and barley in eastern Australia. Australas. Plant Pathol..

[60] Abdallah-Nekache N., Laraba I., Ducos C. (2019). Occurrence of Fusarium head blight and Fusarium crown rot in Algerian wheat: Identification of associated species and assessment of aggressiveness. Eur. J. Plant Pathol..

[61] Chehri K., Salleh B., Yli-mattila T., Soleimani M.J., Yousefi A.R. (2010). Occurrence, pathogenicity and distribution of *Fusarium* spp. in stored wheat seeds Kermanshah Province, Iran. Pak. J. Biol. Sci..

[62] Chehri K. (2011). Occurrence of *Fusarium* species associated with economically important agricultural crops in Iran. Afr. J. Microbiol. Res..

[63] Minati M.H. (2020). First record of nine *Fusarium* spp. causing root rot on wheat (*Triticum aestivum* L.) in Iraq. AIP Conf. Proc..

[64] Fernandez M.R., Jefferson P.G. (2004). Fungal populations in roots and crowns of common and durum wheat in Saskatchewan. Can. J. Plant Pathol..

[65] Rossi V., Cervi C., Chiusa G., Languasco L. (1995). Fungi associated with foot rots on winter wheat in northwest Italy. J. Phytopathol..

[66] Bentley A.R., Cromey M.G., Farrokhi-Nejad R., Leslie J.F., Summerell B.A., Burgess L.W. (2006). Fusarium crown and root rot pathogens associated with wheat and grass stem bases on the South Island of New Zealand. Australas. Plant Pathol..

[67] Jevtić R., Stošić N., Župunski V., Lalošević M., Orbović B. (2019). Variability of stem-base infestation and coexistence of *Fusarium* spp. causing crown rot of winter wheat in Serbia. Plant Pathol. J..

[68] Taheri Esmaeili A., Hamel C., Gan Y., Vujanovic V. (2011). First report of *Fusarium redolens* from Saskatchewan and its comparative pathogenicity. Can. J. Plant Pathol..

[69] Gebremariam E.S., Dababat A.A., Erginbas-Orakci G., Karakaya A., Poudyal D.S., Paulitz T.C. (2016). First report of *Fusarium hostae* causing crown rot on wheat (*Triticum* spp.) in Turkey. Plant Dis..

[70] Kane R.T., Smiley R.W., Sorrells M.E. (1987). Relative pathogenicity of selected *Fusarium* species and *Microdochium bolleyi* to winter wheat in New York. Plant Dis..

[71] Jonavičienė A., Supronienė S., Semaškienė R. (2016). *Microdochium nivale* and *M. majus* as causative agents of seedling blight in spring cereals. Zemdirb.-Agric..

[72] Bouaicha O., Laraba I., Boureghda H. (2022). Identification, in vitro growth and pathogenicity of *Microdochium* spp. associated with wheat crown rot in Algeria. J. Plant Pathol..

[73] Xu F., Shi R.J., Zhang J.J., Song Y.L., Liu L.L., Han Z.H., Wang J.M., Li Y.H., Feng C.H., Li L.J. (2022). First report of *Microdochium nivale* and *M. majus* causing brown foot rot of wheat in China. Plant Dis..

[74] Leyva-Mir S.G., Vega-Portillo H.E., Villaseñor-Mir H.E., Tlapal-Bolaños B., Vargas-Hernández M., Camacho-Tapia M., Tovar-Pedraza J.M. (2017). Characterization of *Fusarium* species causing root rot of wheat in the Bajio, Mexico. Chil. J. Agric. Anim. Sci. Ex Agro-Cienc..

[75] Dehghanpour-Farashah S., Taheri P., Falahati-Rastegar M. (2020). Identification and pathogenicity of *Fusarium* spp., the causal agent of wheat crown and root rot in Iran. J. Plant Pathol..

[76] Tillmann M., von Tiedemann A., Winter M. (2017). Crop rotation effects on incidence and diversity of *Fusarium* species colonizing stem bases and grains of winter wheat. J. Plant Dis. Prot..

[77] Kazan K., Gardiner D.M. (2018). Fusarium crown rot caused by *Fusarium pseudograminearum* in cereal crops: Recent progress and future prospects. Mol. Plant Pathol..

[78] Fernandez M.R., Chen Y. (2005). Pathogenicity of *Fusarium* species on different plant parts of spring wheat under controlled conditions. Plant Dis..

[79] Özer G., Erper I., İmren M., Bozoglu T., Ozdemir F., Dababat A.A. (2022). First report of crown rot caused by *Fusarium algeriense* on wheat in Kyrgyzstan. Plant Dis..

[80] Fouly H.M., Pedersen W.L., Wilkinson H.T., Abd El-Kader M.M. (1996). Wheat root rotting fungi in the “old” and “new” agricultural lands of Egypt. Plant Dis..

[81] Douglas L.I., Deacon J.W. (1994). Strain variation in tolerance of water stress by Idriella *Microdochium bolleyi*, a biocontrol agent of cereal root and stem base pathogens. Biocontrol Sci..

[82] Matušinsky P., Sedláková B., Bleša D. (2022). Compatible interaction of *Brachypodium distachyon* and endophytic fungus *Microdochium bolleyi*. PLoS ONE.

[83] Demirci E., Dane E. (2003). Identification and pathogencity of *Fusarium* spp. from stem bases of winter wheat in Erzurum, Turkey. Phytoparasitica.

[84] Wagacha J.M., Muthomi J.W. (2007). *Fusarium culmorum*: Infection process, mechanisms of mycotoxin production and their role in pathogenesis in wheat. Crop Prot..

[85] Temirbekova S.K., Kulikov I.M., Ashirbekov M.Z., Afanasyeva Y.V., Beloshapkina O.O., Tyryshkin L.G., Zuev E.V., Kirakosyan R.N., Glinushkin A.P., Potapova E.S. (2022). Evaluation of wheat resistance to snow mold caused by *Microdochium nivale* (Fr) Samuels and I.C. Hallett under abiotic stress influence in the Central Non-Black Earth Region of Russia. Plants.

[86] O’Donnell K., Whitaker B.K., Laraba I., Proctor R.H., Brown D.W., Broders K., Geiser D.M. (2022). DNA sequence-based identification of *Fusarium*: A work in progress. Plant Dis..

[87] O’Donnell K., Nirenberg H.I., Aoki T., Cigelnik E.A. (2000). Multigene phylogeny of the *Gibberella fujikuroi* species complex: Detection of additional phylogenetically distinct species. Mycoscience.

[88] Geiser D.M., Al-Hatmi A.M., Aoki T., Arie T., Balmas V., Barnes I., Viljoen A. (2021). Phylogenomic analysis of a 55.1-kb 19-gene dataset resolves a monophyletic *Fusarium* that includes the *Fusarium solani* species complex. Phytopathology.

[89] Li Y.Y., Wang M.M., Groenewald M., Li A.H., Guo Y.T., Wu F., Begerow D. (2022). Proposal of Two New Combinations, Twenty New Species, Four New Genera, One New Family, and One New Order for the Anamorphic Basidiomycetous Yeast Species in Ustilaginomycotina. Front. Microbiol..

[90] Lin L., Pan M., Tian C., Fan X. (2022). Fungal Richness of *Cytospora* Species Associated with Willow Canker Disease in China. J. Fungi.

[91] Liu Q., Wingfield M.J., Duong T.A., Wingfield B.D., Chen S. (2022). Diversity and Distribution of *Calonectria* Species from Plantation and Forest Soils in Fujian Province, China. J. Fungi.

[92] Costa M.M., Saleh A.A., Melo M.P., GuimarŃes E.A., Esele J.P., Zeller K.A., Leslie J.F. (2022). *Fusarium mirum* sp. nov, intertwining *Fusarium madaense* and *Fusarium andiyazi*, pathogens of tropical grasses. Fungal Biol..

[93] Suga H., Kitajima M., Nagumo R., Tsukiboshi T., Uegaki R., Nakajima T., Kushiro M., Nakagawa H., Shimizu M., Kageyama K. (2014). A single nucleotide polymorphism in the translation elongation factor 1alpha gene correlates with the ability to produce fumonisin in Japanese *Fusarium fujikuroi*. Fungal Biol..

[94] Suga H., Arai M., Fukasawa E., Motohashi K., Nakagawa H., Tateishi H., Hyakumachi M. (2019). Genetic differentiation associated with fumonisin and gibberellin production in Japanese *Fusarium fujikuroi*. Appl. Environ. Microbiol..

