# Supplementary material for: Fungal Pathogens Associated with Crown and Root Rot in Wheat-Growing Areas of Northern Kyrgyzstan
Source: J Fungi (Basel). 2023 Jan 16;9(1):124. doi: 10.3390/jof9010124 (PMC9867107; doi:10.3390/jof9010124)
Supplement: Supplementary file 1 [file jof-09-00124-s001.zip › jof-2095094-supplementary.pdf]

Table S1. GenBank Accession numbers of the representative isolates obtained in this study.

Species	GenBank accession numbers			
	**Isolate code**	**ITS**	***TEF1***	***RPB2***
Bipolaris sorokiniana	KrBsor01	OP709681	-	-
	KrBsor02	OP709682	-	-
	KrBsor03	OP709683	-	-
Curvularia ineaqualis	KrCina01	OP709684	-	-
	KrCina02	OP709685	-	-
	KrCina03	OP709686	-	-
Macrophomina phaseolina	KrMpha01	OP709678	-	-
	KrMpha02	OP709679	-	-
	KrMpha03	OP709680	-	-
Microdochium bollleyi	KrMbol01	OP709687	-	-
	KrMbol02	OP709688	-	-
	KrMbol03	OP709689	-	-
Microdochium nivale	KrMniv01	OP709690	-	-
	KrMniv02	OP709691	-	-
	KrMniv03	OP709692	-	-
Fusarium pseudograminearum	KrFpg01	-	OP688131	OP688160
	KrFpg02	-	OP688132	OP688161
	KrFpg03	-	OP688133	OP688162
Fusarium culmorum	KrFcul01	-	OP688134	OP688163
	KrFcul02	-	OP688135	OP688164
	KrFcul03	-	OP688136	OP688165
Fusarium nygamai	KrFnyg01	-	OP688137	OP688166
	KrFnyg02	-	OP688138	OP688167
	KrFnyg03	-	OP688139	OP688168
Fusarium redolens	KrFred01	-	OP688140	OP688169
	KrFred02	-	OP688141	OP688170
	KrFred03	-	OP688142	OP688171
Fusarium oxysporum	KrFoxy01	-	OP688143	OP688172
	KrFoxy02	-	OP688144	OP688173
	KrFoxy03		OP688145	OP688174
Fusarium acuminatum	KrFacu01	-	OP688146	OP688175
	KrFacu02	-	OP688147	OP688176
	KrFacu03	-	OP688148	OP688177
Fusarium equiseti	KrFequ01	-	OP688149	OP688178
	KrFequ02	-	OP688150	OP688179
	KrFequ03	-	OP688151	OP688180
Fusarium proliferatum	KrFpro01	-	OP688152	OP688181
	KrFpro02	-	OP688153	OP688182
Fusarium burgessii	KrFbur01	-	OP688154	OP688183
	KrFbur02	-	OP688155	OP688184
Fusarium tricinctum	KrFtri01	-	OP688156	OP688185
	KrFtri002	-	OP688157	OP688186
Fusarium algeriense	KrFalg01	-	OP688158	OP688187
Fusarium solani	KrFsol01	-	OP688159	OP688188
